# Risk of diaphragmatic hernia in patients with spontaneous pneumothorax

**DOI:** 10.1186/s12890-022-02147-z

**Published:** 2022-09-16

**Authors:** Jian-Xun Chen, Shao-Yun Hsu, Mei-Chen Lin, Pin-Keng Shih

**Affiliations:** 1 School of Medicine, China Medical University, Taichung, Taiwan; 2 Department of Surgery, China Medical University Hospital, No. 2 Yuh-Der Road, Taichung, Taiwan; 3 Division of Reconstructive Microsurgery, Department of Plastic and Reconstructive Surgery, Chang Gung Memorial Hospital, College of Medicine, Chang Gung University, Taoyüan, Taiwan; 4 Management Office for Health Data, China Medical University and Hospital, Taichung, Taiwan

**Keywords:** Spontaneous pneumothorax, Smoking-related disease, Diaphragmatic hernia

## Abstract

**Background:**

Few studies have implied the incidence of diaphragmatic hernia (DH) after spontaneous pneumothorax (SP) with unknown mechanisms. The current study aimed to identify whether there is an association between the DH and SP.

**Methods:**

We selected 46,897 patients with SP (SP cohort) and 46,897 without SP (non-SP matched cohort) from the National Health Insurance Database. Patients were frequency matched according to age, sex, and index year. The incidence of DH and its association with SP were assessed after stratifying different characteristics and comorbidities. Statistical analysis including chi-square test, t-test, cox proportional hazard model, and Kaplan–Meier method were used.

**Results:**

The results suggested there were significant associations between SP and DH, especially in the subgroup of patients with older age (aged 40–64 years: 2.61-fold in adjusted hazard ratio (aHR), 95% confidence interval (CI): 1.27–5.36; aged > 65 years: 1.97-fold in aHR, 95% CI 1.43–2.71), male sex (2.11-fold in aHR, 95% CI 1.56–2.85), hypertension (2.05-fold in aHR, 95% CI 1.30–3.23), diabetes mellitus (2.58-fold in aHR, 95% CI 1.37–4.86), and smoking-related disease (1.86-fold in aHR, 95% CI 1.28–2.71). The SP cohort has significantly correlated with DH within 5-year follow-up (< 2 years: 3.22-fold in aHR, 95% CI 2.10–4.94; 2–5 years: 1.70-fold in aHR, 95% CI 1.05–2.75).

**Conclusions:**

The SP cohort had a higher incidence of DH than the non-SP matched cohort. A prospective study of indications based on the findings of the current research should be performed.

## Introduction

In a global estimate, the incidence of spontaneous pneumothorax (SP) is 7.4–18 cases per 100,000 population each year in males, and 1.2–6 cases per 100,000 population each year in females [1]. In Taiwan, the incidence of SP ranged 5.05 to 7.18 per 100,000 population, and the highest risk was 15–22 years with the incidence rates > 11/100,000 [2]. Most patients with SP manifested pain and dyspnea [3]. The tobacco smoking is a major risk factor for primary SP; chronic obstructive pulmonary disease is most frequently associated with secondary SP [4].

In addition to iatrogenic diaphragmatic hernia (DH) [5, 6], congenital DH is a common structural birth defect that affects approximately 1 in 2500 live births and usually related to lung hypoplasia and persistent pulmonary hypertension [7]. Although the exact mechanism of the congenital DH was not well demonstrated, it is becoming increasingly clear that genetic factors play an important role in many cases of congenital DH [7].

Accumulating data have shown that the SP may be associated with the development of DH [8–10]. The matrix metalloproteinase (MMP) family expression may be a bridge connecting SP and DH [11, 12], but more evidences were still needed. Therefore, the primary outcome in this study is to survey whether there is a significant relationship between SP and DH, and the second outcome is to investigate which variables of the SP cohort made contribution to the significant association.

## Methods

### Data sources

The Taiwan government established the National Health Insurance Research Database (NHIRD), which has a high health coverage rate and included the health claim data of nearly 99% of the Taiwan population from the Taiwan NHI. This study conducted analyses using data from the population-based hospitalization database, which included more than 20 million participants with available data from 1996 to 2013. The identification number of each patient was encrypted to ensure privacy. The diagnoses in the Taiwan NHI are coded according to the International Classification of Disease, Ninth Revision, Clinical Modification (ICD-9-CM). The current study was approved by the research ethics committee of China Medical University and Hospital in Taiwan (CMUH-104-REC2-115-(CR6)) and performed in accordance with the Declaration of Helsinki.

### Study population

To validate the association between SP and DH, we conducted a retrospective cohort study. The participants were classified into the SP and non-SP matched cohorts. Patients with SP who were aged older than 20 years had at least one hospitalization due to SP (ICD-9-CM: 512.0 and 512.8) from 2000 to 2012. The index date was set as the first SP diagnosis date. Subsequently, patients diagnosed with DH before the indexed date or aged < 18 (calculated until the index date) were excluded from this study. The surveying purpose of this study was DH (ICD-9-CM codes 551.3 (diaphragmatic hernia with gangrene), 552.3 (diaphragmatic hernia with obstruction), and 553.3(diaphragmatic hernia)). Patients with at least one hospitalization diagnosis were included. The follow-up period was from the index date to the first diagnosis of DH, withdrawal from the NHIRD, or until December 31, 2013. For each patient with SP, we assigned one who was never diagnosed with SP via propensity score matching. The matching variables were age; sex; index year; and comorbidities including hypertension (ICD-9-CM codes 401–405) [13, 14], diabetes (ICD-9-CM codes 250) [15, 16], hyperlipidemia (ICD-9-CM code 272) [17] and smoking related disease (ICD-9-CM codes 305.1, 430–438, 410–414, 493 and 496) [18].

### Statistical analysis

The categorical variables were sex, age (< 40, 40–64, ≥ 65 years), and comorbidities, and they were presented as numbers and percentages. Age was expressed as mean and standard deviation, and the difference between the two cohorts was assessed using the chi-square test and the t-test.

The association between SP and the risk of DH was evaluated using the cox proportional hazard model. The results were presented as hazard ratios (HR), adjusted hazard ratios (aHR), and 95% confidence intervals (CIs). The Kaplan–Meier method was used to identify the cumulative incidence curves of DH between the SP and non-SP matched cohorts. The difference between two curves was assessed using the log-rank test. All statistical analyses were performed using SAS statistical software version 9.4 (SAS Institute Inc., Cary, NC). The results were plotted using R software. A two-sided *p* value of < 0.05 was considered statistically significant.

## Results

Each cohort comprised 46,897 eligible participants (Table 1). Among them, 80% were men, and the mean age of the participants was 54 years. Approximately 23.3% of patients presented with hypertension, 12.3% with diabetes, 4.2% with hyperlipidemia, and 29.4% with smoking-related disease. All factors did not significantly differ between the two cohorts after propensity score matching (*p* value < 0.05).

**Table 1:** Demographic characteristics and comorbidities of patients newly diagnosed pneumothorax in Taiwan during 2000–2012

Characteristics	Total	Pneumothorax		*p* value
		No N = 46,897	Yes N = 46,897	
Gender				0.994
Female	19,455	9728 (20.7)	9727 (20.7)	
Male	74,339	37,169 (79.3)	37,170 (79.3)	
Age				
< 40	31,670	15,835 (33.8)	15,835 (33.8)	1.000
40–64	21,067	10,535 (22.5)	10,532 (22.5)	
≥ 65	41,057	20,527 (43.8)	20,530 (43.8)	
Mean (SD)^a^				
Baseline comorbidity				
Hypertension	21,883	10,942 (23.3)	10,941 (23.3)	0.994
Diabetes mellitus	11,578	5789 (12.3)	5789 (12.3)	1.000
Hyperlipidemia	3904	1950 (4.2)	1954 (4.2)	0.948
Smoking related diseases	27,613	13,807 (29.4)	13,806 (29.4)	0.994

Chi-square test

^a^t-test

Table 2 shows the association between the potential risk factors and the risk of DH. During the study period, there were 98 SP patients and 120 non-SP patients with DH occurrence. SP (aHR = 2.04, 95% CI 1.55–2.69), older age (aHR = 2.35, 95% CI 1.37–4.05; aHR = 7.14, 95% CI 4.41–11.57), and smoking related disease (aHR = 2.13, 95% CI 1.53–2.96) were found to be associated with a significantly high risk of DH.

**Table 2:** Cox model measured hazard ratio and 95% confidence intervals of diaphragmatic hernia associated with and without Pneumothorax

Characteristics	Event	Univariate		Multivariate	
	(n = 218)	HR (95% CI)	*p* value	HR (95% CI)	*p* value
*Pneumothorax*					
No	120	Ref		Ref	
Yes	98	1.25 (0.95–1.63)	0.106	2.04 (1.55–2.69)	< 0.001
*Gender*					
Female	38	Ref		Ref	
Male	180	1.18 (0.83–1.67)	0.367	1.29 (0.90–1.83)	0.1614
*Age at baseline*					
< 40	24	Ref		Ref	
40–64	31	2.53 (1.48–4.31)	0.0007	2.35 (1.37–4.05)	0.002
≥ 65	163	9.84 (6.40–15.13)	< 0.001	7.14 (4.41–11.57)	< 0.001
*Baseline comorbidity*					
Hypertension	83	3.44 (2.61–4.54)	< 0.001	1.13 (0.81–1.56)	0.477
Diabetes mellitus	40	2.51 (1.78–3.55)	< 0.001	1.18 (0.82–1.70)	0.387
Hyperlipidemia	8	1.23 (0.60–2.48)	0.573	0.51 (0.25–1.05)	0.069
Smoking related diseases	122	5.03 (3.84–6.60)	< 0.001	2.13 (1.53–2.96)	< 0.001

Adjusted HR: adjusted for gender, age, and comorbidities in Cox proportional hazards regression

*HR* Hazard ratio, *CI* Confidence interval

After stratification with variables (Table 3), patients with SP who presented with aged older than 40 years (aged 40–64 years: 2.61-fold in aHR, 95% CI 1.27–5.36; aged > 65 years: 1.97-fold in aHR, 95% CI 1.43–2.71), male (2.11-fold in aHR, 95% CI 1.56–2.85), hypertension (2.05-fold in aHR, 95% CI 1.30–3.23), diabetes mellitus (2.58-fold in aHR, 95% CI 1.37–4.86), and smoking-related disease (1.86-fold in aHR, 95% CI 1.28–2.71) had a significantly higher risk of DH.

**Table 3:** Incidence rates, hazard ratio and confidence intervals of diaphragmatic hernia in different stratification

Variables	Matched cohort			Pneumothorax			HR			
	n = 46,897			n = 46,897			Crude	*p* value	Adjusted	*p* value
	Event	Person years	IR	Event	Person years	IR	(95% CI)		(95% CI)	
*Age at baseline*										
< 40	10	110,528	0.90	14	106,107	1.32	1.46 (0.65–3.29)	0.360	1.51 (0.67–3.41)	0.318
40–64	13	69,029	1.88	18	39,739	4.53	2.39 (1.17–4.89)	0.017	2.61 (1.27–5.36)	0.009
≥ 65	97	104,241	9.31	66	36,345	18.16	1.85 (1.34–2.53)	< 0.001	1.97 (1.43–2.71)	< 0.001
*Gender*										
Female	24	58,723	4.09	14	33,679	4.16	1.00 (0.52–1.93)	0.997	1.75 (0.89–3.46)	0.105
Male	96	225,076	4.27	84	148,512	5.66	1.30 (0.97–1.74)	0.081	2.11 (1.56–2.85)	< 0.001
*Comorbidity*										
Hypertension	51	50,045	10.19	32	15,815	20.23	1.89 (1.20–2.97)	0.006	2.05 (1.30–3.23)	0.002
Diabetes mellitus	22	26,633	8.26	18	8852	20.33	2.43 (1.29–4.56)	0.006	2.58 (1.37–4.86)	0.004
Hyperlipidemia	4	9431	4.24	4	3614	11.07	2.78 (0.69–11.26)	0.151	3.21 (0.78–13.24)	0.107
Smoking related diseases	76	66,553	11.42	46	22,646	20.31	1.71 (1.18–2.48)	0.005	1.86 (1.28–2.71)	0.001

Adjusted HR: adjusted for gender, age, and comorbidities in Cox proportional hazards regression

*IR* Incidence rates, per 10,000 person-years, *HR* Hazard ratio, CI Confidence interval

When stratified by follow-up years, the SP cohort who were followed up for less than fivers years from the index date showed a significantly higher risk of DH (< 2 years: 3.22-fold in aHR, 95% CI 2.10–4.94; 2–5 years: 1.70-fold in aHR, 95% CI 1.05–2.75) (Table 4).

**Table 4:** Incidence rates, hazard ratio and confidence intervals of diaphragmatic hernia in different follow-up stratification

Follow-up years	Matched cohort			Pneumothorax			HR			
	n = 46,897			n = 46,897			Crude	*p* value	Adjusted	*p* value
	Event	Person years	IR	Event	Person years	IR	(95% CI)		(95% CI)	
< 2	35	88,122	3.97	57	61,511	9.27	2.30 (1.51–3.50)	< 0.001	3.22 (2.10–4.94)	< 0.001
2–5	46	96,660	4.76	28	59,589	4.70	0.98 (0.62–1.57)	0.946	1.70 (1.05–2.75)	0.033
> 5	39	99,017	3.94	13	61,091	2.13	0.84 (0.69–1.02)	0.056	1.05 (0.55–2.03)	0.876

Adjusted HR: adjusted for gender, age, and comorbidities in Cox proportional hazards regression

*IR* Incidence rates, per 10,000 person-years, HR Hazard ratio, CI Confidence interval

**Fig. 1:**
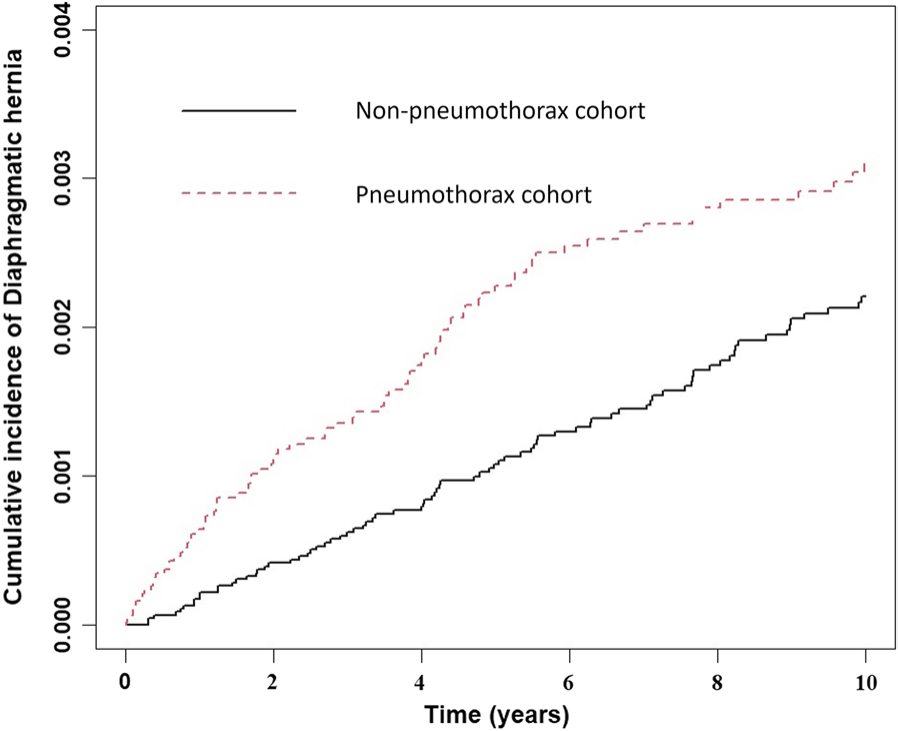
The pneumothorax group had a significantly higher cumulative incidence of diaphragmatic hernia than the non-pneumothorax group (log-rank test; *p* = 0.003)

The SP cohort had a significantly higher cumulative incidence of DH than the non-SP group in Fig. 1 (log-rank test; *p* = 0.003).

## Discussion

The results in this study found the SP cohort had a significantly higher cumulative incidence of DH than the non-SP group in the 10-year follow-up. The risk factors of DH include SP, older age, and smoking related disease. After stratification with various subgroup, the SP cohort with older age (aged > 40 years), male sex, hypertension, diabetes mellitus, and smoking-related disease had signification associations with the DH.

There may be controversies regarding whether the DH was congenital or caused by trauma. To prevent bias, the patients with DH who had a previous history of trauma within the last 5 years were not included to prevent including those suspected with congenital DH.

This study showed that the SP cohort had a higher incidence of DH than the non-SP matched cohort, one proposed mechanism might be attributed to possible collagen fiber defect which resulted from systemic collagenase effect [7, 19]. Although there were lack of direct evidences, previous studies had showed that MMP-9 might play an important role in the stability of collagen fiber development at molecular level [20]. Moreover, Masumoto, K. et al. found a clear increase in MMP-1 but decrease in TIMP-2-reactive capillaries and fibroblasts in congenital DH tissues, which emphasize the essential role of MMP family in collagen structure [11]. Yet, owing to lack of well-established mechanism, further molecular level studies are warranted to identify the connection of collagen structure in SP and DH.

There were significant associations between SP and DH in the subgroup of patients with older age (aged > 40 years), male sex, hypertension, diabetes mellitus, and smoking-related disease. (Table 3). A previous study showed that smoking, older age, diabetes mellitus and hypertension can increase systemic soft tissue remodeling [20–22]. Therefore, our findings can be explained by the fact that the systemic predisposition of SP was strengthened by the abovementioned risk factors. By contrast, Wen CP et al. showed that the male:female smoker ratio was 10.9:1 among Taiwanese adults. Moreover, the prevalence of smoking was substantially high during high school years, and it peaked at age 30–39 years [23]. Hence, the high incidence of DH in older male patients with SP (> 40 years) may be associated with smoking.

Our data suggested the SP was significantly correlated with DH within 5-year follow-up, but not in follow-up longer than 5 years (Table 4). The detailed mechanisms were not well demonstrated, but we suspect the phenomenon may be attributed to a balance between proteolysis and anti-proteolysis achieved in longer follow-up. Further research was necessary.

To the best of our knowledge, this is the first large-scale and long-term follow-up study showing the association between SP and DH. At the end of the 10-year follow-up, the SP cohort had a significantly higher cumulative incidence of DH than the non-SP matched cohort (Fig. 1). Because the SP patients with the comorbidities such as hypertension, diabetes mellitus, and smoking-related disease were significantly with DH, we suspected the SP patients with medicine control for hypertension and diabetes mellitus as well as decreased smoking exposure may lessen the incidence of DH.

The current study had several limitations. First, data about lifestyles factors such as smoking habits, socioeconomic status, drug history, and genetic factors were not obtained for adjusting the risk of DH due to data bank limitation. Second, although the diagnoses of DH were based on admission diagnoses and codes provided by surgeons, an extremely precise analysis of data about patients without admission and severity/grades was not performed. Third, because all data used were anonymized, relevant clinical variables, such as surgical findings, imaging results, and laboratory data, were not available. Fourth, patients with DH who had a previous history of trauma within the last 5 years were excluded. Therefore, its incidence might have been overestimated. Fifth, our data bank duration ranged from 1996 to 2013, which is not really reflective of the latest situation. Sixth, the apparent higher incidence of DH in SP group might be attributed to difference in follow up assessment, healthcare seeking behavior, loss to follow-up, variation in coding practice, etc. Further research was suggested. Seventh, the biases associated with the retrospective nature of the study might have existed.

## Conclusion

In summary, the SP cohort, particularly patients with older age (aged > 40 years), male sex, hypertension, diabetes mellitus, and smoking-related disease, had a higher incidence of DH than the non-SP matched cohort. In addition, the SP was significantly correlated with DH within 5-year follow-up. A prospective study of indications based on the findings of the current research should be performed.

## Data Availability

This study used inpatient claims data from the Taiwan National Health Insurance Research Database (NHIRD). This database contains detailed medical histories of the hospitalized enrollees in Taiwan. Based on the guideline of Taiwan Ministry of Health and Welfare (TMHW), only citizens of the Taiwan are eligible to apply the NHIRD for research projects (https://nhird.nhri.org.tw/en/Data_Protection.html). The database we applied is only limited to our research purpose. All applicants must follow the Computer-Processed Personal Data Protection Law and related regulations of National Health Insurance Administration and NHRI (https://nhird.nhri.org.tw/en/Data_Files.html). The ownership of NHIRD is belong to TMHW and the right to use is belong to the researchers. However, other researchers (under the permission of CMUH-104-REC2-115-CR6, China Medical University and Hospital) are able to request data access following the regulations of TMHW.
